# Supplementary figures and images for: Drug resistance and its risk factors among extrapulmonary tuberculosis in Ethiopia: A systematic review and meta-analysis
Source: PLoS One. 2021 Oct 8;16(10):e0258295. doi: 10.1371/journal.pone.0258295 (PMC8500428; doi:10.1371/journal.pone.0258295)

Literature search strategy


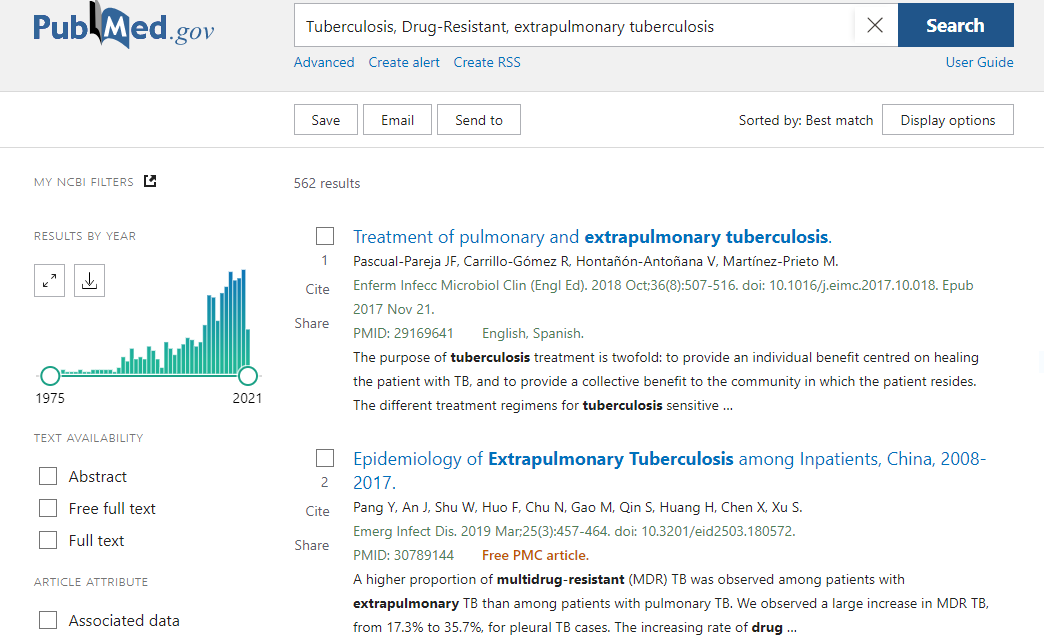


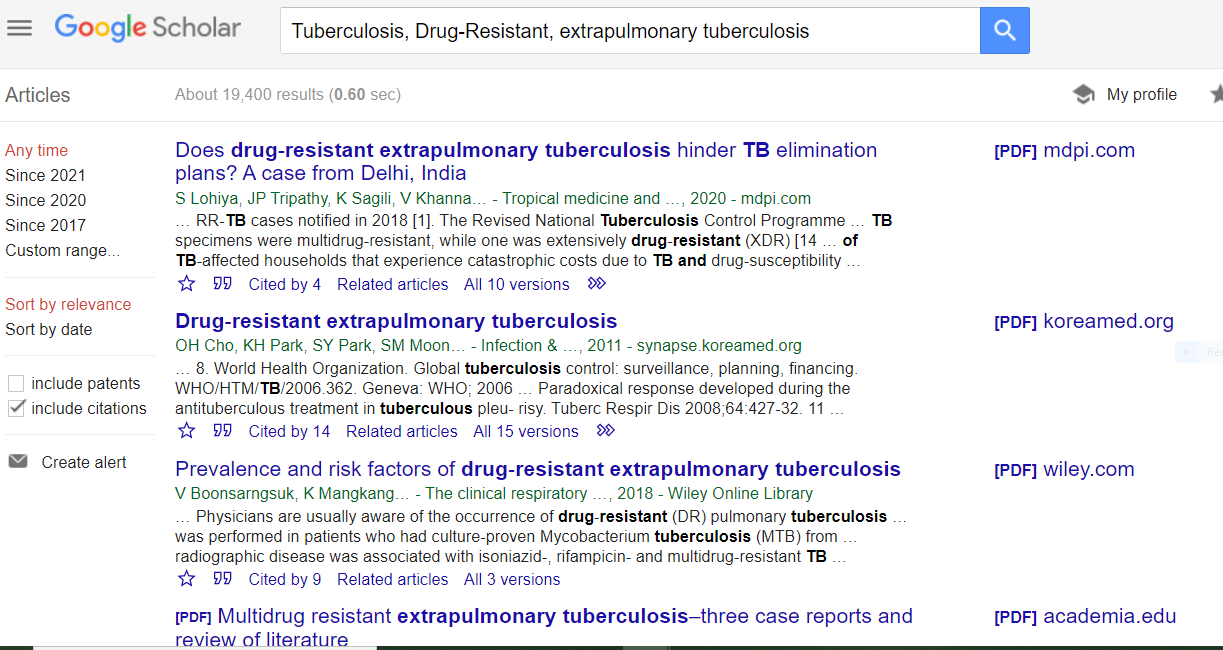


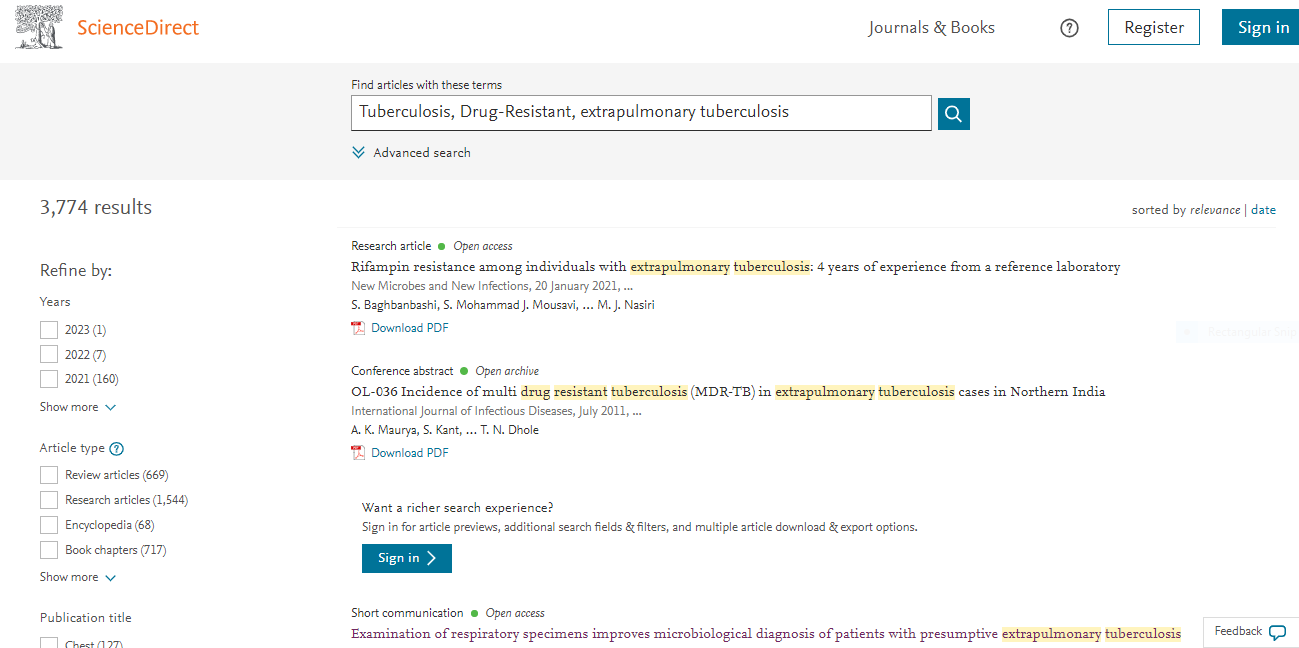

Supplement: S1 File — (DOCX) [file pone.0258295.s001.docx]
