# Supplementary material for: Drug resistance and its risk factors among extrapulmonary tuberculosis in Ethiopia: A systematic review and meta-analysis
Source: PLoS One. 2021 Oct 8;16(10):e0258295. doi: 10.1371/journal.pone.0258295 (PMC8500428; doi:10.1371/journal.pone.0258295)
Supplement: S4 File — (DOCX) [file pone.0258295.s004.docx]

Newcastle-Ottawa quality assessment scale for cross sectional studies

| Author, year | Q1 | | | Q2 | | | Q3 | | | Q4 | | | Q5 | | | Q6 | | | Q7 | | | Q8 | | | Q9 | | | Q10 | | | Overall quality result |
| --- | --- | --- | --- | --- | --- | --- | --- | --- | --- | --- | --- | --- | --- | --- | --- | --- | --- | --- | --- | --- | --- | --- | --- | --- | --- | --- | --- | --- | --- | --- | --- |
|  | Yes | No | Can’t tell | Yes | No | Can’t tell | Yes | No | Can’t tell | Yes | No | Can’t tell | Yes | No | Can’t tell | Yes | No | Can’t tell | Yes | No | Can’t tell | Yes | No | Can’t tell | Yes | No | Can’t tell | Yes | No | Can’t tell |  |
| Biadglegne et al. 2014 | √ |  |  | √ |  |  | √ |  |  |  |  | √ | √ |  |  | √ |  |  | √ |  |  | √ |  |  | √ |  |  | √ |  |  | 90% |
| Mulu et al  2017 | √ |  |  | √ |  |  | √ |  |  | √ |  |  |  | √ |  |  | √ |  | √ |  |  | √ |  |  | √ |  |  | √ |  |  | 80% |
| Bekele et al 2018 | √ |  |  | √ |  |  | √ |  |  | √ |  |  |  | √ |  |  | √ |  | √ |  |  | √ |  |  | √ |  |  | √ |  |  | 80% |
| Tadesse et al  2015 | √ |  |  | √ |  |  | √ |  |  | √ |  |  |  | √ |  |  | √ |  | √ |  |  | √ |  |  | √ |  |  | √ |  |  | 80% |
| Korma et al 2015 | √ |  |  | √ |  |  | √ |  |  | √ |  |  |  | √ |  |  | √ |  | √ |  |  | √ |  |  | √ |  |  | √ |  |  | 80% |
| Zewdie et al 2017 | √ |  |  | √ |  |  | √ |  |  | √ |  |  |  | √ |  |  | √ |  | √ |  |  | √ |  |  | √ |  |  | √ |  |  | 80% |
| Sitotaw et al 2017 | √ |  |  | √ |  |  | √ |  |  |  | √ |  |  | √ |  |  | √ |  | √ |  |  | √ |  |  | √ |  |  | √ |  |  | 70% |
| Diriba et al  2020 | √ |  |  | √ |  |  | √ |  |  |  |  | √ | √ |  |  | √ |  |  | √ |  |  | √ |  |  | √ |  |  | √ |  |  | 90% |

****Y=yes, N=nor, NA=not applicable, <60%=low, 60-80%=medium, >80%=high quality***
