# Supplementary material for: Drug resistance and its risk factors among extrapulmonary tuberculosis in Ethiopia: A systematic review and meta-analysis
Source: PLoS One. 2021 Oct 8;16(10):e0258295. doi: 10.1371/journal.pone.0258295 (PMC8500428; doi:10.1371/journal.pone.0258295)
Supplement: Attachment — Submitted filename: Comments for Authers.docx [file pone.0258295.s005.docx]

**Comments for Authors**

1	The date of electronic database searching is not mentioned in method or else, maybe it need updating electronic database searching.

2.	On fig 1, out of 195 studies 136 studies were excluded in which 59 were eligible for full text

evaluation, but the authors wrote 60.

3.	Lack of consistency of using words through out of the manuscript for example in line 34, 37 ‘extrapulmonary but in line 54 extra-pulmonary

Further proofread and copyediting are required for this manuscript. The paper must be carefully checked by a native English speaker.
