# Supplementary material for: Drug resistance and its risk factors among extrapulmonary tuberculosis in Ethiopia: A systematic review and meta-analysis
Source: PLoS One. 2021 Oct 8;16(10):e0258295. doi: 10.1371/journal.pone.0258295 (PMC8500428; doi:10.1371/journal.pone.0258295)
Supplement: Attachment — Submitted filename: Response to Reviewers.docx [file pone.0258295.s006.docx]

**Ref: PONE-D-21-12541**

**Drug resistance and its risk factors among extrapulmonary tuberculosis in Ethiopia: A systematic review and meta-analysis**

Dear Editor,

Thank you for your informative comments and thoughtful comments on our manuscript. We have addressed your and the reviewers comments one by one. We also appreciate the reviewers for their critical observation and informative comments which radically improved our manuscript quality. We have provided point-by-point response to the reviewers' comments below. Also, we would like to inform you that we have used hyperlink to indicate where we made changes in the previous version of the manuscript based on the reviewers’ comments.

**Editor Comments to the Author**

Comment #1: Please ensure that your manuscript meets PLOS ONE's style requirements, including those for file naming. The PLOS ONE style templates can be found at

**Response:** Thank you for your feedback. The support files have been updated based on the PLOS ONE style.

Comment #2: In your Methods, please state the timeline of your literature search.

**Response:** Thank you for your suggestions and constructive comments. To address this comment, we have indicated the timeline of our literature search period (line 105-106).

Comment #3: Please include captions for your Supporting Information files at the end of your manuscript, and update any in-text citations to match accordingly. Please see our Supporting Information guidelines for more information: http://journals.plos.org/plosone/s/supporting-information.

**Response:** Thank you very much for your informative comments. We have addressed your comments by adding captions for the supporting files after the references (Page # 21).

**Review Comments to the Author**

Please use the space provided to explain your answers to the questions above. You may also include additional comments for the author, including concerns about dual publication, research ethics, or publication ethics. (Please upload your review as an attachment if it exceeds 20,000 characters)

**Reviewer #1:**

Comment #1: The date of electronic database searching is not mentioned in method or else, maybe it needs updating electronic database searching.

**Response:** Thank you very much for your critical observation and we apologize for not indicating the timeline for the literature search. To address this comment, we have added the timeline of our literature search since we were recorded the time of our search period (Line 105-106).

Comment #2: On fig 1, out of 195 studies 136 studies were excluded in which 59 were eligible for full text evaluation, but the authors wrote 60.

**Response:** Thank you for your insightful comments and we apologize for the error. We have corrected the errors in fig 1 based on your comments.

Comment #3: Lack of consistency of using words through out of the manuscript for example in line 34, 37 ‘extrapulmonary but in line 54 extra-pulmonary

Further proofread and copyediting are required for this manuscript. The paper must be carefully checked by a native English speaker.

**Response:** We have used the words consistently across the document and the language usage and grammar related errors have been corrected by native English speaker colleagues.

**Reviewer #2:**

 Comment #1: Literature Searches and Search terms are incomplete. This is suboptimal for publication for systematic review. Please attach search terms that were used in each database as supplement for Data source and search strategies in the manuscript. Please provide details search terms in supplementary documents. Please attach syntax used in each database as supplementary. Authors should also search Embase in their study.

**Response:** Thank you for your critical observations and informative comments. We have attached our search strategy for the databases we used during our search and those that support advanced search as supporting file name "S1: File". For example, advanced search for Google scholar cannot be supported and we just used PICO based on the title only. You are absolutely right, EMBASE should be searched. However, since EMBASE is not freely accessed in our setup, we could not search it. We believe that the majority of the articles published from Ethiopia are on MEDLINE/PubMed, which can be accessed through PubMed search. Thus, the articles that would be missed by our search are small.

Comment #2: When Pubmed is used for the search, MESH terms are always recommended to be included.

**Response:** Indeed, we apologize for not indicating as we used MESH term during PubMed search. To address this comment, we have added the phrase which indicates that we used MESH term in our search strategy (Line 110).

Comment #3: Evaluation of gray literature is unclear.

**Response**: We have evaluated the quality of gray literature as any article published as a peer reviewed article. However, we included only one gray literature, which could not seriously affect the quality of critical appraisal.

Comment #4: Please report here the process of search and inclusion/exclusion of the study and the reasons of exclusion in detail. Inclusion and exclusion criteria should be better detailed (e.g., epidemiological study design).

**Response:** Thank you very much for your critical observation. We have addressed your comment by elaborating the inclusion and exclusion criteria of the study in detail (line 113-121).

Comment #5: The method for selecting studies is not clear and need further explanation.

**Response:** The PRISMA flow chart clearly indicates the study selection process of our review. In addition to the PRISMA flow chart, the text we have provided on page # 6; lines 123 – 130 under the subheading study selection clearly indicated our study selection process. To address your comment, we have elaborated on our selection criteria during inclusion and exclusion criteria provision.

Comment #6: Quality assessments for all included papers should be shown as a supplementary file.
**Response:** Thank you for your insightful comments and suggestions. Based on your comments, we have corrected and attached the quality results of each included study as the supplementary file.

Comment #7: Why authors used "Random effects model". More details are needed to explain the statistical plan.

**Response:** The random-effects model was used because of the heterogeneity of the true effect sizes of the included studies. Thus, we believe the explanation provided is enough for the readers. Interested body can read reference books that explain the random-effects model.

Comment #8: Applying egger weighted regression method was stated in statistical methods but was not reported in result.

**Response:** We apologize for not reporting the result of publication bias in the previous version of our manuscript. To address this comment, we have added the results of the funnel plot and Egger test to show the presence of publication bias (Line196-199; page # 9)

Comment #9: There is substantive heterogeneity in outcomes, which the authors have reported but have not done anything. The random-effects model is not a good solution for a high source of heterogeneity. The author can conduct a meta-analysis in sub-groups and report the possible sources of heterogeneity.

**Response:** Yes, you are right to conduct sub-group analysis to assess the reason for heterogeneity. However, since the studies included in this review were few, they cannot allow us to conduct sub-group analysis based on several factors. Moreover, the random-effects model is the recommended model when there is heterogeneity between the true effect sizes, as we have indicated in the statistical analysis part of the manuscript.

Comment #10: To investigate the publication bias, a funnel plot has been used. Since this plot and other methods of evaluation, the publication bias in this study is based on the value of the effect size and the standard error, and in descriptive studies, there is no effect size. What do
they represent?

**Response:** The study specific and pooled results are presented on the forest plots (Fig 2 to Fig 4). Thus, reporting standard errors and effect sizes separately are meaningless.

Yours, Sincerely

Getu Diriba
